# Association of Maternal Opioid Use in Pregnancy With Adverse Perinatal Outcomes in Ontario, Canada, From 2012 to 2018

**DOI:** 10.1001/jamanetworkopen.2020.8256

**Published:** 2020-07-29

**Authors:** Daniel J. Corsi, Helen Hsu, Deshayne B. Fell, Shi Wu Wen, Mark Walker

**Affiliations:** 1 OMNI Research Group, Clinical Epidemiology Program, Centre for Practice Changing Research, Ottawa Hospital Research Institute, Ottawa, Ontario, Canada; 2 School of Epidemiology and Public Health, University of Ottawa, Ottawa, Ontario, Canada; 3 Children’s Hospital of Eastern Ontario Research Institute, Ottawa, Ontario, Canada; 4 Department of Family Medicine, University of Ottawa, Ottawa, Ontario, Canada; 5 Department of Obstetrics and Gynecology, University of Ottawa, Ottawa, Ontario, Canada

## Abstract

**Importance:**

A recent epidemic of opioid abuse has been described in many communities, although population-based data on trends in use in pregnancy and perinatal outcomes after in utero exposure remain limited.

**Objective:**

To assess trends in prenatal opioid use and the potential association between prenatal opioid use and preterm birth and adverse perinatal outcomes.

**Design, Setting, and Participants:**

This population-based retrospective cohort study covered live births and stillbirths among adolescents and women 15 years and older from April 1, 2012, to March 31, 2018, in Ontario, Canada. Data were analyzed from July 29 to October 15, 2019.

**Exposures:**

Any opioid use in pregnancy, ascertained through self-reporting and routine prenatal care.

**Main Outcome and Measures:**

The primary outcome was preterm birth before a gestational age of 37 weeks. Separate indicators for birth occurring at gestational ages of 34 to 36 weeks (plus 6 to 7 days; late preterm), 32 to 33 weeks (plus 6 to 7 days), 28 to 31 weeks (plus 6 to 7 days), and less than 28 weeks (very preterm birth). Secondary outcomes included small for gestational age, stillbirth, transfer to neonatal intensive care, and 5-minute Apgar score. Coarsened exact matching techniques and Poisson regression models were used to estimate the risk difference and relative risk (RR) of outcomes associated with cannabis exposure to control for confounding.

**Results:**

Among 710 911 women included in the analytic sample (mean [SD] age, 30.4 [5.3] years), 8059 used opioids (1.1%), with prevalence decreasing from 1.31% (95% CI, 1.25%-1.38%) in fiscal year 2012-2013 to 1.05% (95% CI, 0.99%-1.11%) in fiscal year 2017-2018 (*P* < .001 for trend). Use was highest among women in the lowest quintile of area-level income (2.36% vs 0.56% in the highest quintile; RR, 3.86; 95% CI, 3.58-4.15) and did not decrease over time in this group (from 2.63% [95% CI, 2.41%-2.87%] in 2012-2013 to 2.35% [95% CI, 2.14%-2.58%] in 2017-2018; *P* = .23 for trend). The crude rate of preterm birth at a gestational age of less than 37 weeks was 14.0% (n = 1127) among women with reported use in pregnancy and 6.0% (n = 42 226) among women who did not use opioids in the unmatched cohort. The adjusted RR for preterm birth before a gestational age of 37 weeks was 1.63 (95% CI, 1.52-1.75) among opioid users compared with nonusers and 1.77 (95% CI, 1.35-2.31) for preterm birth before 32 weeks. Among newborns, risk for neonatal intensive care was 40.5% with perinatal exposure to opioids compared with 13.9% in unexposed infants (RR, 2.91; 95% CI, 2.80-3.03).

**Conclusions and Relevance:**

Rates of opioid use in pregnancy have declined in recent years, although use remains significantly higher among lower-income women. In this large population-based cohort, opioid use in pregnancy was associated with an increased risk of preterm birth and admission to a neonatal intensive care unit.

## Key Points

**Question:**

What are the trends in opioid use in pregnancy and is there an association between prenatal opioid use and perinatal outcomes?

**Findings:**

In this cohort study that included 804 346 births, 8059 women reported opioid use, and the rate of opioid use declined from 1.31% in 2012 to 2013 to 1.05% in 2017 to 2018. Use was higher among lower socioeconomic status groups and was associated with a 60% increase in risk of preterm birth before a gestational age of 37 weeks (14.0% among women with opioid use vs 6.0% in those with no use).

**Meaning:**

These findings suggest that opioid use in pregnancy is associated with higher rates of preterm birth and admission to neonatal intensive care after adjustment for confounding factors, and that rates of use have declined in recent years.

## Teaser

This cohort study assesses trends in prenatal opioid use and the potential association between prenatal opioid use and preterm birth and adverse perinatal outcomes in a population-based perinatal database in Ontario, Canada.

## Introduction

Rates of hospitalization and death due to opioid overdose have increased significantly during the last decade in the United States and Canada.^1,2,3^ Concurrent rates of opioid use and misuse in pregnancy have been increasing, with corresponding increases in maternal and neonatal morbidity.^4,5,6,7^ In Ontario, a 16-fold increase has occurred in the number of infants born to women with opioid dependence, increasing from 46 to 800 per year from 2002 to 2014.^8^ The incidence of neonatal abstinence syndrome (NAS), which can occur in neonates exposed to opioids in utero,^9,10^ has increased during the past 2 decades in Ontario, from 0.28 per 1000 live births in 1992 to 4.29 per 1000 live births in 2011,^11^ reflecting the increase in prenatal use.^12,13,14^ Previous research, beginning with work by Finnegan in the 1970s^15^ and replicated more recently in the United States and elsewhere, indicates an association between intrauterine exposure to opioids and low birth weight,^15,16,17^ small for gestational age (SGA),^18,19^ and preterm birth.^19,20,21^ These infants have higher rates of admission to neonatal intensive care units (NICUs), placing a considerable economic burden on the health care system.^22^

Opioids, as a class of drugs, cover natural derivatives of the opium poppy as well as endogenous and synthetic compounds. Opioids are fundamentally pain relievers, but the associated euphoria and dissociation often lead to recreational use, addiction, and dependence.^23^ Among the many forms are illicit use (eg, heroin) and use associated with opioid agonist maintenance treatment (eg, methadone hydrochloride or buprenorphine hydrochloride combined with naloxone hydrochloride). In pregnancy, women with an opioid use disorder can undergo opioid agonist maintenance treatment to reduce complications in pregnancy associated with withdrawal and improve outcomes in mother-infant dyads.^24,25^ Buprenorphine is a potential first-line treatment to improve neonatal outcomes in this population.^23^ Compared with methadone-exposed neonates, buprenorphine-exposed neonates require less morphine sulfate to treat NAS and have shorter hospital stays.^26^ Buprenorphine also appears to be associated with lower overall NAS scores.^27,28^

At present, population-based data are lacking regarding trends in opioid use in pregnancy and the effects of prenatal exposure on maternal and neonatal outcomes in Canada. Two studies^29,30^ have described the incidence of prenatal opioid use and rates of NAS in Northwestern Ontario. Opioid use has increased in that region, although neonatal outcomes have improved owing to the implementation of a family medicine–based prenatal and obstetric program.^30^ Given the potential perinatal health and social effects on mothers and their children, it is essential to describe these associations in the Canadian context. We used the Better Outcomes Registry and Network (BORN), a comprehensive province-wide perinatal registry in the province of Ontario and the largest perinatal database in Canada,^31^ to conduct a population-based study of trends in maternal opioid use in pregnancy and associated perinatal outcomes.

## Methods

Research ethics board approval for this study was obtained from the Ottawa Health Science Network Research Ethics Board and the Children’s Hospital of Eastern Ontario. As a prescribed registry, BORN has the authorization to collect, use, and disclose personal health information without consent to facilitate or improve the provision of health care. We followed the Strengthening the Reporting of Observational Studies in Epidemiology (STROBE) reporting guideline to report the methods underlying this observational cohort study.

### Study Population and Data Source

The BORN Ontario birth registry captures pregnancies and births occurring in Ontario and represents about 40% of all births in Canada.^31^ Data collection in BORN includes information on maternal demographic characteristics, health behaviors (including substance use), preexisting health problems, obstetric complications, intrapartum events, birth outcomes, and admission to neonatal intensive care. Data in the birth registry are routinely collected from medical records, clinical forms, and patient interviews when a woman is admitted to hospital for birth. A continuous program of data quality checks is in place, and formal training sessions are provided to maintain the accuracy of the registry.^31^ A validation study of 29 variables found more than 90% agreement between the registry and patient medical records for more than three-quarters of the audited variables, with the remaining variables demonstrating fair-to-moderate agreement.^32^ We extracted a retrospective cohort from the BORN registry consisting of women who delivered a singleton infant at a gestational age of 20 or more weeks with a birth weight of 500 g or more in an Ontario hospital from April 1, 2012, to March 31, 2018.

### Maternal, Obstetric, Perinatal, and Neonatal Outcomes

The primary outcome was preterm birth at a gestational age of less than 37 weeks. Preterm birth is a critical indicator of perinatal health^33^ and a significant risk factor for infant morbidity and mortality.^22^ Separate binary indicators were defined for births at a gestational age of less than 37 weeks (all preterm births), 34 to 36 weeks (late preterm), less than 32 weeks, and less than 28 weeks (very preterm birth). Secondary perinatal outcomes were SGA at birth (<10th percentile and <3rd percentile), birth weight, and incidence of stillbirth. Neonatal outcomes included transfer to a NICU and a 5-minute Apgar score of less than 4.^34,35^

### Exposure

Information on maternal use of opioids in pregnancy, including medications for opioid use disorder, was collected during routine prenatal care for mothers. All pregnant women completed standardized perinatal records with their obstetrician, family physician, or midwife at the first prenatal consultation, where substance use in the current pregnancy was recorded. The median gestational age for the initial prenatal consultation was 11 (interquartile range, 8-16) weeks. Also, nurses can identify opioid use during clinical histories obtained from patients at admission to the hospital for labor and delivery. Exposure was defined as any opioid use in the current pregnancy, including illicit use, prescribed use, or opioid agonist treatment. We evaluated the accuracy of opioid use coding in the birth registry by reviewing and comparing the antenatal and delivery records of a sample of 100 patients, with the medical records considered the criterion standard. A random sample of patients was selected and stratified by year, maternal age, and diagnosis of NAS in the infant. Substance use history was abstracted from medical records by trained medical record abstractors who were blinded to the opioid coding in the birth registry. Sensitivity, specificity, and positive and negative predictive values were calculated.

### Covariates

Maternal age at delivery was derived from maternal birth date and date of delivery. Area-level income quintiles and urban or rural residential location were assigned from the Canadian census.^36^ We linked postal codes using the Postal Code Conversion File Plus, version 6, developed by Statistics Canada.^37^ Through this linkage, we obtained area-level income information based on the census. Parity, antenatal care clinician (family physician, obstetrician, or midwife), year of birth, smoking, alcohol exposure, use of cannabis, use of cocaine, and use of other substances were included as covariates.

### Statistical Analysis

Data were analyzed from July 29 to October 15, 2019. Women with reported use of opioids during pregnancy were compared with those with no use across baseline characteristics using standardized differences.^38^ Standardized differences are a comparison of means of the covariates across opioid users and nonusers, presented in units of the pooled SD.^39^ Unlike conventional statistical tests, the standardized difference is not influenced by sample size; we use this metric to examine differences across groups.

We used coarsened exact matching methods to reduce confounding and imbalance in the data across sociodemographic and obstetric characteristics between opioid users and nonusers.^40^ Specifically, we matched opioid users and nonusers within defined categories of covariates.^30^ This process involved 2 steps before statistical analyses. First, age, parity, area-level income quintile, smoking, alcohol use, cannabis use, cocaine use, other drug use, antenatal care clinician, year of birth, and rural residence were categorized as presented in Table 1. Next, opioid users and nonusers were matched in a ratio of 1:n within strata representing unique combinations of covariate categories, using weights to normalize the distribution of nonusers to equal the number of users. Any strata with 0 opioid users or 0 nonusers were excluded. The remaining data formed the matched cohort, and, within this cohort, we compared the distribution of covariates between opioid users and nonusers with standardized differences.

**Table 1.** Characteristics of Women With and Without Opioid Use in Pregnancy in the Unmatched and Matched Cohorts^a^

Characteristic	Unmatched sample			Matched sample^b^		
	Opioid use, No. (%)		SMD	Opioid use, No. (%)		SMD
	No (n = 702 852)	Yes (n = 8059)		No (n = 529 058)	Yes (n = 6502)	
Age, y						
15-19	25 949 (3.7)	496 (6.2)	0.39	9499 (5.8)	375 (5.8)	<0.001
20-24	95 997 (13.7)	2061 (25.6)		66 208 (24.8)	1612 (24.8)	
25-29	222 661 (31.7)	2697 (33.5)		178 983 (33.7)	2193 (33.7)	
30-34	239 555 (34.1)	1938 (24.0)		194 488 (24.9)	1616 (24.9)	
≥35	118 690 (16.9)	867 (10.8)		79 880 (10.9)	706 (10.9)	
Parity (not including index pregnancy)						
0	302 835 (43.1)	2575 (32.0)	0.42	238 485 (33.5)	2179 (33.5)	<0.001
1	247 266 (35.2)	2349 (29.1)		198 157 (30.0)	1952 (30.0)	
2	100 528 (14.3)	1554 (19.3)		64 246 (18.8)	1223 (18.8)	
≥3	52 223 (7.4)	1581 (19.6)		28 170 (17.7)	1148 (17.7)	
Area-level income quintile						
1	106 933 (15.2)	2581 (32.0)	0.51	78 628 (30.7)	1996 (30.7)	<0.001
2	109 229 (15.5)	1644 (20.4)		80 021 (20.3)	1318 (20.3)	
3	145 748 (20.7)	1497 (18.6)		107 585 (18.8)	1220 (18.8)	
4	166 834 (23.7)	1350 (16.8)		128 440 (17.3)	1127 (17.3)	
5	174 108 (24.8)	987 (12.2)		134 384 (12.9)	841 (12.9)	
Substance use in current pregnancy^c^						
Tobacco	52 787 (7.5)	4664 (57.9)	1.27	31 475 (51.8)	3365 (51.8)	<0.001
Alcohol	15 638 (2.2)	751 (9.3)	0.31	1475 (4.6)	300 (4.6)	<0.001
Cannabis	9221 (1.3)	1126 (14.0)	0.49	1755 (8.2)	536 (8.2)	<0.001
Cocaine	837 (0.1)	554 (6.9)	0.37	47 (0.7)	45 (0.7)	<0.001
Antenatal care						
Family physician	179 077 (25.5)	3236 (40.2)	0.51	127 320 (40.6)	2638 (40.6)	<0.001
Obstetrician	409 710 (58.3)	3585 (44.5)		350 396 (47.2)	3071 (47.2)	
Midwife	100 079 (14.2)	535 (6.6)		48 854 (6.5)	422 (6.5)	
Other/none	13 986 (2.0)	703 (8.7)		2488 (5.7)	371 (5.7)	
Year of birth (fiscal year)						
2012-2013	113 487 (16.1)	1509 (18.7)	0.08	90 975 (19.8)	1290 (19.8)	<0.001
2013-2014	117 390 (16.7)	1423 (17.7)		92 838 (18.8)	1221 (18.8)	
2014-2015	113 375 (16.1)	1246 (15.5)		81 380 (15.4)	999 (15.4)	
2015-2016	119 058 (16.9)	1297 (16.1)		87 106 (15.5)	1005 (15.5)	
2016-2017	119 962 (17.1)	1315 (16.3)		90 980 (15.6)	1012 (15.6)	
2017-2018	119 580 (17.0)	1269 (15.7)		85 779 (15.0)	975 (15.0)	
Residence in rural area	95 261 (13.6)	1851 (23.0)	0.25	37 144 (21.6)	1404 (21.6)	<0.001

Abbreviation: SMD, standardized mean difference.

^a^ Data are from the Ontario Better Outcomes Registry and Network perinatal database from fiscal years 2012-2013 to 2017-2018. Percentages have been rounded and may not total 100.

^b^ In the matched sample, unweighted counts and weighted percentages are reported.

^c^ Indicates reported use of substances in current pregnancy. Tobacco use is any reported use at admission to labor and delivery. Alcohol use is any reported use during current pregnancy.

We fit Poisson regression models with robust sandwich variance estimators^41,42^ for associations between prenatal opioid exposure and outcomes. Poisson models with robust variance estimation are an alternative to logistic regression for the analyses of binary outcomes in cohort studies with the advantage of directly estimating the relative risk.^43,44^ A term for fiscal year (from April 1 to March 31) was included to assess linear trends. Models were adjusted for the matching factors to account for any residual confounding within strata. Associations were reported as relative risks (RRs) and associated 95% CIs and compared with crude analyses conducted in the unmatched cohort for each outcome.

We conducted subgroup analyses and tests of statistical interaction using 2-sided Wald tests in the matched cohort. We examined potential effect modification in the association of opioid use on preterm birth in subgroups of women identified a priori who smoked, used alcohol, used cocaine, or used cannabis during pregnancy. Statistical tests were 2 sided with the criterion for statistical significance set at α = .05. All analyses were conducted in R, version 3.5.2 (R Project for Statistical Computing).

## Results

The initial cohort consisted of 804 346 pregnancies resulting in a singleton birth. We excluded 39 982 women (5.0%) who were missing opioid use information and an additional 53 453 observations (6.6%) with missing covariate data, yielding a final analytic sample of 710 911. The mean (SD) gestational age of infants was 39 (2) weeks, 51.4% were male, and 48.6% were female. Mothers had a mean (SD) age of 30.4 (5.3) years, and 8059 (1.1%) reported any illicit or prescribed opioid use. An analysis of the excluded records indicated some modest differences by age (standardized mean difference [SMD], 0.21), area-level income (SMD, 0.19), antenatal care (SMD, 0.42), year of birth (SMD, 0.20), and rural residence (SMD, 0.17) (eTable 1 in the Supplement). In a sample of 100 randomly selected maternal-infant dyads, including 51 with a diagnosis of NAS in the infants, 19 mothers had opioid use recorded in clinical records of antenatal or delivery encounters. The remaining 81 patients had no recorded use. All 19 infants prenatally exposed to opioids had a diagnosis of NAS. Thirty-two additional infants with NAS had exposure to other substances, including cocaine or amphetamines. Compared with clinical records, opioid use documented in the birth registry had a sensitivity of 84% (95% CI, 60%-97%) and a specificity of 98% (95% CI, 91%-99%).

We identified an imbalance in the distribution of covariates between women with opioid use and those with no use. Standardized mean differences of greater than 0.10 existed for maternal age (SMD, 0.39), parity (SMD, 0.42), area-level income (SMD, 0.51), maternal tobacco use (SMD, 1.27), alcohol use (SMD, 0.31), cannabis use (SMD, 0.49), cocaine use (SMD, 0.37), antenatal care clinician (SMD, 0.51), and residence in a rural area (SMD, 0.25). The coarsened exact matching procedure removed imbalance in measured baseline covariates between those with and without reported opioid use (all SMDs <0.001). The matched study cohort consisted of pregnancies in 535 560 women, of whom 6502 had reported opioid use (Table 1).

### Trends

**Figure 1.**
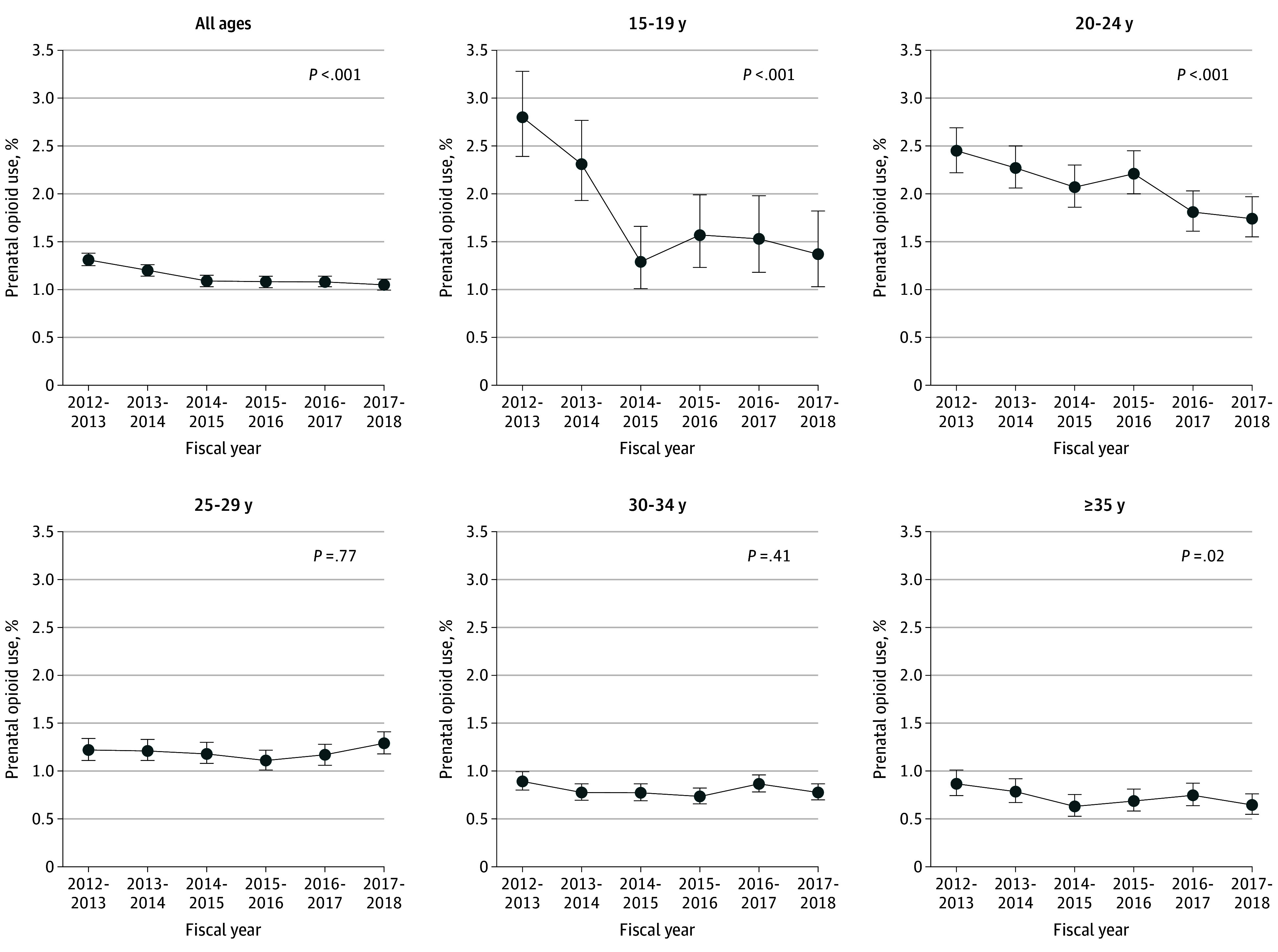
Trends in Rate of Prenatal Opioid Use by Maternal Age Group Data are from the Better Outcomes Registry and Network Ontario perinatal database from fiscal years 2012-2013 to 2017-2018. *P* values are calculated for linear trend. Whiskers represent 95% CIs.

The rate of opioid use in pregnancy in Ontario declined from 1.31% (95% CI, 1.25%-1.38%) in fiscal year 2012-2013 to 1.05% (95% CI, 0.99%-1.11%) in fiscal year 2017-2018 (*P* < .001 for trend). From 2012-2013 to 2017-2018, the relative decrease in opioid use was 16% (RR, 0.84; 95% CI, 0.78-0.90) with adjusting for maternal age, area-level income, and rural residence. Prenatal opioid use was more prevalent among women younger than 25 years (Figure 1). Declines in rates of opioid use were variable by age group (F_4,710 901_ = 9.86; *P* < .001). Among women aged 15 to 19 years, the prevalence of prenatal opioid use was 2.80% (95% CI, 2.39%-3.28%) in 2012-2013, declining to 1.37% (95% CI, 1.03%-1.82%) in 2017-2018 (*P* < .001 for trend), a relative decrease of 51% (RR, 0.49; 95% CI, 0.35-0.68). In 2012-2013, the prevalence of prenatal opioid use was 2.45% (95% CI, 2.22%-2.69%) among women aged 20 to 24 years, decreasing to 1.74% (95% CI, 1.55%-1.97%) in 2017-2018 (*P* < .001 for trend). A decline in use was also observed among women 35 years and older, from 0.87% (95% CI, 0.75%-1.01%) in 2012-2013 to 0.65% (95% CI, 0.55%-0.76%) in 2017-2018 (*P* = .02 for trend), equivalent to a relative reduction of 25% (RR, 0.75; 95% CI, 0.59-0.93).

**Figure 2.**
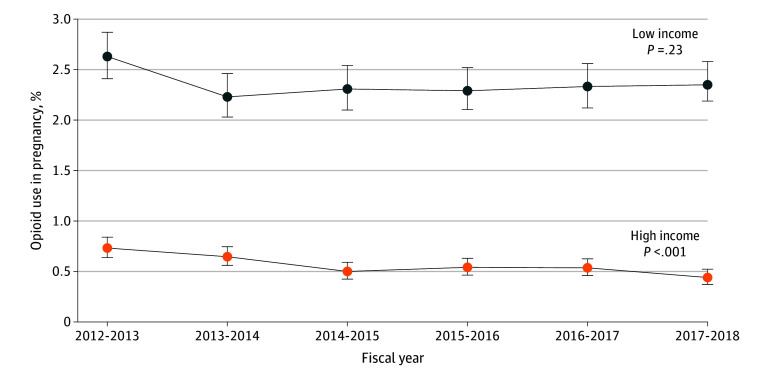
Trends in Rate of Prenatal Opioid Use in Ontario by Low- and High-Income Groups According to Maternal Area-Level Income Classification Data are from the Better Outcomes Registry and Network Ontario perinatal database from fiscal years 2012-2013 to 2017-2018. *P* values are calculated for linear trend. Whiskers represent 95% CIs.

Across all years, area-level income was strongly associated with prenatal opioid use. The RR was 3.86 (95% CI, 3.58-4.15) for use of opioids among women in quintile 1 (lowest income; 2.36%) compared with women in quintile 5 (highest income; 0.56%). Heterogeneity in opioid use trends was seen across area-level income quintiles (F_4,710 901_ = 2.95; *P* = .02) (Figure 2). Among women in quintile 5 (highest income), the rate of opioid use was 0.73% (95% CI, 0.64%-0.84%) in 2012-2013, decreasing to 0.44% (95% CI, 0.37-0.52) in 2017-2018 (*P* < .001 for trend), a relative decrease of 40% (RR, 0.60; 95% CI, 0.48-0.75). In quintile 1 (lowest income), the rate of use was 2.63% (95% CI, 2.41%-2.87%) in 2012-2013 and 2.35% (95% CI, 2.14%-2.58%) in 2017-2018 (*P* = .23 for trend). Compared with residence in urban areas, residence in rural areas was associated with increased opioid use in pregnancy (RR, 2.04; 95% CI, 1.94-2.15). Declines were found in urban areas, from 1.18% (95% CI, 1.11%-1.24%) in 2012-2013 to 0.93% (95% CI: 0.87-0.99) in 2017-2018 (*P* < .001), and rural areas, from 2.26% (95% CI, 2.03%-2.52%) in 2012-2013 to 1.78% (95% CI, 1.59%-1.99%) in 2017-2018 (*P* = .006). A test of interaction revealed no statistical difference in the rate of change across the area of residence (*P* = .60) (eFigure in the Supplement).

### Association Between Prenatal Opioid Use and Preterm Birth

The crude rate of preterm birth at a gestational age of less than 37 weeks was 14.0% (n = 1127) among women with reported use in pregnancy and 6.0% (n = 42 226) among women who did not use opioids in the unmatched cohort (unadjusted risk difference [RD], 7.97%; 95% CI, 7.21%-8.73%) (Table 2). The risk of preterm birth was higher among women with reported prenatal opioid use for all categories of gestational age. However, absolute risks of early preterm birth at gestational age of less than 32 weeks were lower in both groups owing to fewer events (no use, 5236 of 702 852 [0.7%]; opioid use, 156 of 8059 [1.9%]). Opioid exposure was also associated with increases in rates of secondary perinatal and neonatal outcomes, including SGA (3rd percentile and 10th percentile), admission to a NICU, and 5-minute Apgar scores of less than 4, compared with women with no opioid use in the unmatched cohort. For instance, the rate of SGA (3rd percentile) was 4.6% (n = 373) in infants exposed to opioids compared with 2.4% (n = 17 121) among those without exposure (unadjusted RD, 2.19%; 95% CI, 1.73%-2.65%).

**Table 2.** Pregnancy Outcomes in Women With and Without Opioid Use, With Risk Difference and Relative Risk Associated With Cannabis Exposure in the Unmatched Cohort^a^

Outcome	No. of events (risk, %)		RD (95% CI), %^b^	RR (95% CI)^b^
	No opioid use (n = 702 852)	Opioid use (n = 8059)		
Preterm birth, gestational age, wk				
<37	42 226 (6.0)	1127 (14.0)	7.97 (7.21-8.73)	2.33 (2.20-2.46)
34-36 + 6 d	32 590 (4.7)	830 (10.7)	6.00 (5.31-6.70)	2.28 (2.13-2.43)
32-33 + 6 d	4373 (0.7)	141 (2.0)	1.32 (0.99-1.65)	3.03 (2.56-3.60)
28-31 + 6 d	3200 (0.5)	104 (1.5)	1.00 (0.70-1.29)	3.07 (2.51-3.75)
<28	2063 (0.3)	52 (0.7)	0.43 (0.22-0.63)	2.39 (1.80-3.18)
Perinatal outcomes				
SGA (<3rd percentile)	17 121 (2.4)	373 (4.6)	2.19 (1.73-2.65)	1.90 (1.72-2.10)
SGA (<10th percentile)	65 760 (9.4)	1173 (14.6)	5.21 (4.43-5.98)	1.56 (1.47-1.64)
Stillbirth	1890 (0.3)	39 (0.5)	0.21 (0.06-0.37)	1.80 (1.31-2.47)
Neonatal outcomes				
Transfer to NICU	82 700 (11.8)	3531 (43.8)	32.02 (30.93-33.10)	3.73 (3.63-3.82)
5-min Apgar score <4^c^	4183 (0.6)	93 (1.2)	0.55 (0.32-0.78)	1.94 (1.58-2.38)

Abbreviations: NICU neonatal intensive care unit; RD, risk difference; RR, relative risk; SGA small for gestational age.

^a^ Data are from the Ontario Better Outcomes Registry and Network perinatal database from fiscal years 2012-2013 to 2017-2018.

^b^ Adjusted for infant sex. Confidence intervals account for repeated pregnancies within mothers.

^c^ Scores range from 0 to 10; lower scores indicate depressed vitality.

In matched analyses, maternal use of opioids was associated with an RD of 4.97% (95% CI, 4.20%-5.74%) and an RR of 1.63 (95% CI, 1.52-1.75) for preterm birth at a gestational age of less than 37 weeks. Maternal use of opioids was associated with an increased risk for preterm birth at gestational ages of 34 to 36 weeks (RD, 3.86% [95% CI, 3.16%-4.57%]; RR, 1.63 [95% CI, 1.50-1.77]), 32 to 33 weeks (RD, 0.83% [95% CI, 0.50%-1.16%]; RR, 1.87 [95% CI, 1.51-2.31]), 28 to 31 weeks (RD, 0.51% [95% CI, 0.24%-0.79%]; RR, 1.77 [95% CI, 1.35-2.31]), and less than 28 weeks (RD, 0.25% [95% CI, 0.04%-0.45%]; RR, 1.61 [95% CI, 1.15-2.26]) (Table 3).

**Table 3.** Pregnancy Outcomes in Women With and Without Opioid Use, With RD and RR Associated With Cannabis Exposure in the Matched Cohort^a^

Outcome	No. of events (risk, %)		RD (95% CI), %^b^	RR (95% CI)^b^
	No opioid use (n = 529 058)	Opioid use (n = 6502)		
Preterm birth, gestational age, wk				
<37	31 463 (7.8)	829 (12.7)	4.97 (4.20 to 5.74)	1.63 (1.52 to 1.75)
34-36 (+ 6 d)	24 300 (6.0)	619 (9.8)	3.86 (3.16 to 4.57)	1.63 (1.50 to 1.77)
32-33 (+ 6 d)	3245 (1.0)	105 (1.8)	0.83 (0.50 to 1.16)	1.87 (1.51 to 2.31)
28-31 (+ 6 d)	2402 (0.7)	67 (1.2)	0.51 (0.24 to 0.79)	1.77 (1.35 to 2.31)
<28	1516 (0.4)	38 (0.7)	0.25 (0.04 to 0.45)	1.61 (1.15 to 2.26)
Perinatal outcomes				
SGA (<3rd percentile)	13 056 (3.6)	265 (4.1)	0.46 (−0.01 to 0.93)	1.13 (1.00 to 1.27)
SGA (<10th percentile)	50 701 (11.8)	887 (13.6)	1.81 (1.01 to 2.62)	1.15 (1.09 to 1.23)
Stillbirth	1372 (0.3)	26 (0.4)	0.11 (−0.03 to 0.24)	1.38 (0.97 to 1.98)
Neonatal outcomes				
Transfer to NICU	61 977 (13.9)	2635 (40.5)	26.58 (25.48 to 27.69)	2.91 (2.80 to 3.03)
5-min Apgar score <4^c^	3036 (0.7)	62 (1.0)	0.22 (0.01 to 0.43)	1.37 (1.07 to 1.74)

Abbreviations: NICU neonatal intensive care unit; RD, risk difference; RR, relative risk; SGA small for gestational age.

^a^ Data are from the Ontario Better Outcomes Registry and Network perinatal database from fiscal years 2012-2013 to 2017-2018. Cohort was matched on all Table 1 variables using coarsened exact matching.

^b^ Adjusted for infant sex. Standard errors account for repeated pregnancies within mothers.

^c^ Scores range from 0 to 10; lower scores indicate depressed vitality.

After matching, rates of SGA below the 3rd percentile were slightly higher among infants with prenatal exposure to opioids compared with those without (risk, 4.1% vs 3.6%), as were those below the 10th percentile (risk, 13.6% vs 11.8%, respectively). Compared with the matched analyses, the unadjusted RD for SGA below the 10th percentile associated with prenatal opioid use was 5.21% (95% CI, 4.43%-5.98%), decreasing to 1.81% (95% CI, 1.01%-2.62%) after matching. The unadjusted RD for SGA below the 3rd percentile was 2.19% (95% CI, 1.73%-2.65%) in crude analyses and 0.46% (95% CI, −0.01% to 0.93%) after matching. In the matched sample, the mean (SD) birth weight was 3206.3 (616.7) g in infants of women using opioids in pregnancy compared with 3301.2 (571.5) g in infants without opioid exposure (RD, −94.9% [95% CI, −108.8% to −81.0%] g) (eTable 2 in the Supplement). Stillbirth was not associated with prenatal opioid use after matching. Transfer to the NICU was strongly associated with opioid use in pregnancy (risk of 40.5% vs 13.9%). The RD for transfer to the NICU was 26.58% (95% CI, 25.48%-27.69%), and the RR was 2.91 (95% CI, 2.80-3.03) in the matched analyses. An Apgar score of less than 4 at 5 minutes was slightly more frequent among neonates with prenatal exposure to opioids (risk, 1.0% vs 0.7%). The RD was 0.22% (95% CI, 0.01%-0.43%), and the RR was 1.37 (95% CI, 1.07-1.74).

We conducted a comparison of the RDs and RRs of prenatal opioid use on preterm birth at a gestational age of less than 37 weeks by subgroups of women who reported use of tobacco, alcohol, opioids, or no other substances in pregnancy (eTable 3 in the Supplement). Among women who reported use of opioids but no other substances, the crude rate of preterm birth was 12.9% (391 of 3028) compared with 5.8% (36 510 of 634 315) among women who reported no use of substances. In the matched cohort, the RD for this comparison was 6.88% (95% CI, 5.74%-8.01%), and the RR was 2.18 (95% CI, 1.99-2.40).

## Discussion

The study we report here is, to our knowledge, one of the most extensive population-based studies of opioid use in pregnancy in Canada. We have 3 principal findings with relevance to the ongoing opioid epidemic. First, we found that the prevalence of opioid use in pregnancy in Ontario was relatively stable, approximately 1%, but it has declined slightly since 2012. Second, rates of opioid use in pregnancy were higher among younger women younger than 25 years and women of lower socioeconomic status based on area-level income. In particular, women from the lowest 2 quintiles of area-level income represented more than half of the women with reported opioid use in this population. Third, we found clear associations between prenatal opioid use and preterm birth and transfer to a NICU. These associations remained robust after matching across important covariates and potential confounding factors.

Previous studies from Ontario show increasing rates of NAS from 1992 to 2011^11^ and an increasing number of infants born to mothers with opioid dependence from 2002 to 2014.^8^ An examination of the annual changes in the later study suggest that the number of infants born to mothers with opioid dependence was stable from 2012 to 2014 after showing large increases from 2002 to 2012.^8^ Rising rates of NAS are of public health interest owing to complications in newborns and requirements for neonatal intensive care. Our data indicate that rates of prenatal opioid use have further stabilized and even declined since 2012, particularly among younger women and among groups with higher socioeconomic status. At the same time, the rate of transfer to neonatal intense care was stable during the study period. These data suggest that although progress is being made to address rates of opioid use in pregnancy, some groups remain at higher risk, including mothers at lower levels of socioeconomic status in whom rates of opioid use are as much as 5 times higher.

Prenatal opioid use was associated with an increased risk of preterm birth, and the association was consistent across all subcategories in the matched cohort. We did not find a graded association across subcategories of preterm birth, and the highest RR was for preterm birth at a gestational age of 32 to 33 weeks (plus 6 days). The lack of a marked increase in the association across subcategories of preterm birth suggests that although exposure to opioids may be associated with preterm birth, the risk was similar for early and late preterm birth. This association contrasts with other risk factors for preterm birth, which may show graded increases at earlier gestational ages.^45,46^ In subgroup analyses, the association with preterm birth at a gestational age of less than 37 weeks appeared stronger among women with opioid use but without any other reported substance use, suggesting a lack of synergistic effects with other substances. Small for gestational age (<3rd percentile) and stillbirth were only weakly associated with opioid use during pregnancy, and these associations were less consistent in terms of RD. Our finding was smaller in magnitude compared with that of a Danish population-based study^19^ with a prevalence ratio of 1.5 (95% CI, 1.0-2.3) for SGA after accounting for tobacco use. A Swedish study, however, failed to find a statistically significantly increased association between buprenorphine treatment and SGA.^21^ Both studies did not have data available on the association with stillbirth, and it is not clear whether models were adjusted for potential confounders.

### Limitations

This study has some limitations. First, in the BORN Ontario birth registry, maternal opioid use in pregnancy may be underidentified.^47^ Our medical record audit indicated that the capture of maternal opioid exposure in the BORN database was accurate compared with medical records, although both sources have similar biases and do not record all prescribed medications. We did not have access to complete data on NICU treatment, limiting further analyses of infants requiring intensive care. Another data limitation was the lack of complete data on specific sociodemographic variables, such as race/ethnicity. Second, the exposure includes prescribed opioids for analgesia as well as illicit use. Also, women with addiction and opioid use disorder are often prescribed opioid agonist treatments.^48,49^ Stigma associated with substance use and fear of intervention by child protection services may influence reporting patterns, particularly for illicit use.^50,51,52^ The prevalence of prenatal opioid use was similar to the Canadian Tobacco, Alcohol and Drugs Survey (2017), which shows that about 1.7% of girls and women aged 12 to 44 years reported past-year use of opioid pain relievers to get high.^53^ Third, many opioid-dependent women receive opioid-agonist treatments, such as methadone or buprenorphine, during pregnancy. Although owing to data limitations, we did not differentiate between forms of opioids and opioid-agonist therapy in these analyses, the literature suggests that buprenorphine, methadone, and other opioids may be associated with a higher frequency of preterm birth compared with the overall population.^19,21^ Preliminary data from Ontario suggest that compared with opioid maintenance therapy, the sociodemographic and medical/mental health profiles of women with long-term use of prescription opioids are similar, which supports treatment as a single exposure (Astrid Guttmann, MDCM, MSC; email communication; March 27, 2020). Among women with an opioid use disorder, however, medications such as buprenorphine may mitigate some of the risk and improve neonatal outcomes.^26^ The effects of underreporting and exposure misclassification may attenuate associations toward the null hypothesis, making associations observed herein potentially more conservative. Fourth, when describing the prevalence of opioid use over time, we assumed a linear trend. Although alternate specifications such as segmented regression may result in marginal improvement in model fit, we did not specify a priori years when a change in trend may be occurring. Finally, prenatal opioid use cannot be studied as a randomized intervention, and studies relying on observational data are at risk for confounding. To address this, we used matching to create a balanced data set between women with opioid use and those without across available covariates and potential confounders. Residual differences may remain in other measured and unmeasured confounders and partly explain associations with perinatal outcomes.

## Conclusions

In summary, this work provides a detailed assessment of recent trends in opioid use in pregnancy among women in Ontario and the association of opioid use with perinatal and neonatal outcomes. Overall rates of opioid use in pregnancy were stable, although visible gradients exist by socioeconomic status. Rates of opioid use were significantly higher among women of lower socioeconomic status and may be declining among women of higher socioeconomic status. Further research is needed to identify patterns of use and associated maternal-infant outcomes for different classes of opioids. Among pregnant women in this large population cohort, overall opioid use was significantly associated with an increased risk of preterm birth and the need for neonatal intensive care.

## Notes

Supplement. **eTable 1.** Comparison Between Excluded Participants and the Study Population Across Selected Covariates, BORN Ontario Birth Registry, Fiscal Years 2012-2013 to 2017-2018 **eTable 2.** Mean Birth Weight in Infants of Mothers With and Without Prenatal Exposure to Opioids, Matched and Unmatched Cohorts, BORN Ontario Birth Registry, Fiscal Years 2012-2013 to 2017-2018 **eTable 3.** Subgroup Analysis of the Association of Opioid Use on Preterm Birth < 37 Weeks’ Gestation Stratified by Tobacco Use, Alcohol Use, Cocaine Use, and Cannabis Use in the Matched Cohort, BORN Ontario Birth Registry, Fiscal Years 2012-2013 to 2017-2018 **eFigure.** Trends in the Rate of Prenatal Opioid Use in Ontario in Urban and Rural Areas, Fiscal Years 2012-2013 to 2017-2018
