# Supplementary material for: Association of Maternal Opioid Use in Pregnancy With Adverse Perinatal Outcomes in Ontario, Canada, From 2012 to 2018
Source: JAMA Netw Open. 2020 Jul 29;3(7):e208256. doi: 10.1001/jamanetworkopen.2020.8256 (PMC12064095; doi:10.1001/jamanetworkopen.2020.8256)
Supplement: Supplement. — eTable 1. Comparison Between Excluded Participants and the Study Population Across Selected Covariates, BORN Ontario Birth Registry, Fiscal Years 2012-2013 to 2017-2018 eTable 2. Mean Birth Weight in Infants of Mothers With and Without Prenatal Exposure to Opioids, Matched and Unmatched Cohorts, BORN Ontario Birth Registry, Fiscal Years 2012-2013 to 2017-2018 eTable 3. Subgroup Analysis of the Association of Opioid Use on Preterm Birth < 37 Weeks’ Gestation Stratified by Tobacco Use, Alcohol Use, Cocaine Use, and Cannabis Use in the Matched Cohort, BORN Ontario Birth Registry, Fiscal Years 2012-2013 to 2017-2018 eFigure. Trends in the Rate of Prenatal Opioid Use in Ontario in Urban and Rural Areas, Fiscal Years 2012-2013 to 2017-2018 [file jamanetwopen-e208256-s001.pdf]

Supplementary Online Content

Corsi DJ, Hsu H, Fell DB, Wen SW, Walker M. Association of maternal opioid use in pregnancy with adverse perinatal outcomes in Ontario, Canada, from 2012 to 2018. *JAMA Netw Open*. 2020;3(7):e208256. doi:10.1001/jamanetworkopen.2020.8256

eTable 1. Comparison Between Excluded Participants and the Study Population Across Selected Covariates, BORN Ontario Birth Registry, Fiscal Years 2012-2013 to 2017-2018
eTable 2. Mean Birth Weight in Infants of Mothers With and Without Prenatal Exposure to Opioids, Matched and Unmatched Cohorts, BORN Ontario Birth Registry, Fiscal Years 2012-2013 to 2017-2018
eTable 3. Subgroup Analysis of the Association of Opioid Use on Preterm Birth < 37 Weeks' Gestation Stratified by Tobacco Use, Alcohol Use, Cocaine Use, and Cannabis Use in the Matched Cohort, BORN Ontario Birth Registry, Fiscal Years 2012-2013 to 2017-2018
eFigure. Trends in the Rate of Prenatal Opioid Use in Ontario in Urban and Rural Areas, Fiscal Years 2012-2013 to 2017-2018

This supplementary material has been provided by the authors to give readers additional information about their work.

©2020 Corsi DJ et al. *JAMA Network Open*.

eTable 1. Comparison Between Excluded Participants and the Study Population Across Selected Covariates, BORN Ontario Birth Registry, Fiscal Years 2012-2013 to 2017-2018

	**Excluded (N=93,435)**		**Study population (N=710,911)**		
	**n**	**%**	**n**	**%**	**SMD**
Age, years					0.21
15-19	3,332	(3.6)	26,445	(3.7)	
20-24	9,834	(10.5)	98,058	(13.8)	
25-29	24,253	(26.0)	225,358	(31.7)	
30-34	34,315	(36.7)	241,493	(34.0)	
35+	21,701	(23.2)	119,557	(16.8)	
Parity (not including index pregnancy)					0.06
0	38,458	(45.9)	305,410	(43.0)	
1	28,161	(33.6)	249,615	(35.1)	
2	11,119	(13.3)	102,082	(14.4)	
3+	6,118	(7.3)	53,804	(7.6)	
Area-level income quintile					0.19
1	10,968	(13.0)	109,514	(15.4)	
2	10,838	(12.9)	110,873	(15.6)	
3	15,500	(18.4)	147,245	(20.7)	
4	19,188	(22.8)	168,184	(23.7)	
5	27,810	(33.0)	175,095	(24.6)	
Substance use in current pregnancy					
Tobacco	5,660	(10.0)	57,451	(8.1)	0.07
Alcohol	1,248	(2.6)	16,389	(2.3)	0.02
Cannabis	1,362	(2.8)	10,347	(1.5)	0.09
Cocaine	462	(0.9)	1,391	(0.2)	0.10
Antenatal care					0.42
Family physician	11,097	(17.3)	182,313	(25.6)	
Obstetrician	45,988	(71.6)	413,295	(58.1)	
Midwife	3,527	(5.5)	100,614	(14.2)	
Other/none	3,582	(5.6)	14,689	(2.1)	
Year of birth					0.20
2012/13	19,812	(21.2)	114,996	(16.2)	
2013/14	14,637	(15.7)	118,813	(16.7)	
2014/15	19,066	(20.4)	114,621	(16.1)	
2015/16	13,493	(14.4)	120,355	(16.9)	
2016/17	13,037	(14.0)	121,277	(17.1)	
2017/18	13,390	(14.3)	120,849	(17.0)	
Residence in rural area	7,890	(8.4)	97,112	(13.7)	0.17

©2020 Corsi DJ et al. *JAMA Network Open*.

eTable 2. Mean Birth Weight in Infants of Mothers With and Without Prenatal Exposure to Opioids, Matched and Unmatched Cohorts, BORN Ontario Birth Registry, Fiscal Years 2012-2013 to 2017-2018

	**Unmatched Cohort**			**Matched Cohort**		
	**No Opioid Use (N=702,852)**	**Opioid use (N=8,059)**	**Risk Difference, % (95% CI)**	**No Opioid Use (N=529,058)**	**Opioid use (N=6,502)**	**Risk Difference, % (95% CI)**
Birth weight, Mean (SD)						
	3374.6 (544.3)	3173.4 (626.9)	-201.2 (-213.1, 189.2)	3301.2 (571.5)	3206.3 (616.7)	-94.9 (-108.8, -81.0)
Risk difference adjusted for infant sex. Matched cohort accounts for age, parity, area-level income, smoking, alcohol, cannabis, and other drug use in pregnancy, antenatal care provider. year of birth, and residence in a rural area.						

©2020 Corsi DJ et al. *JAMA Network Open*.

eTable 3. Subgroup Analysis of the Association of Opioid Use on Preterm Birth < 37 Weeks' Gestation Stratified by Tobacco Use, Alcohol Use, Cocaine Use, and Cannabis Use in the Matched Cohort, BORN Ontario Birth Registry, Fiscal Years 2012-2013 to 2017-2018

**Subgroup**	**Risk Difference (95% CI)**			**P-interaction**	**Relative Risk (95% CI)**			**P-interaction**
Tobacco use	3.34	-0.25 to	6.93	<0.001	1.35	(0.99 to	1.84)	<0.001
No Tobacco use	6.63	5.54 to	7.73		2.10	(1.91 to	2.30)	
Alcohol use	1.35	-8.10 to	10.80	0.07	1.16	(0.42 to	3.16)	0.12
No Alcohol use	5.09	4.28 to	5.90		1.65	(1.54 to	1.78)	
Cocaine use	-			0.06	-			0.02
No Cocaine use	5.02	4.25 to	5.80		1.65	(1.54 to	1.77)	
Cannabis use	2.83	-12.95 to	18.61	0.22	1.20	(0.44 to	3.23)	0.003
No Cannabis use	5.12	4.33 to	5.91		1.71	(1.59 to	1.83)	
**Opioid use only**	6.88	5.74 to	8.01	-	2.18	(1.99 to	2.40)	-

©2020 Corsi DJ et al. *JAMA Network Open*.

eFigure. Trends in the Rate of Prenatal Opioid Use in Ontario in Urban and Rural Areas, Fiscal Years 2012-2013 to 2017-2018

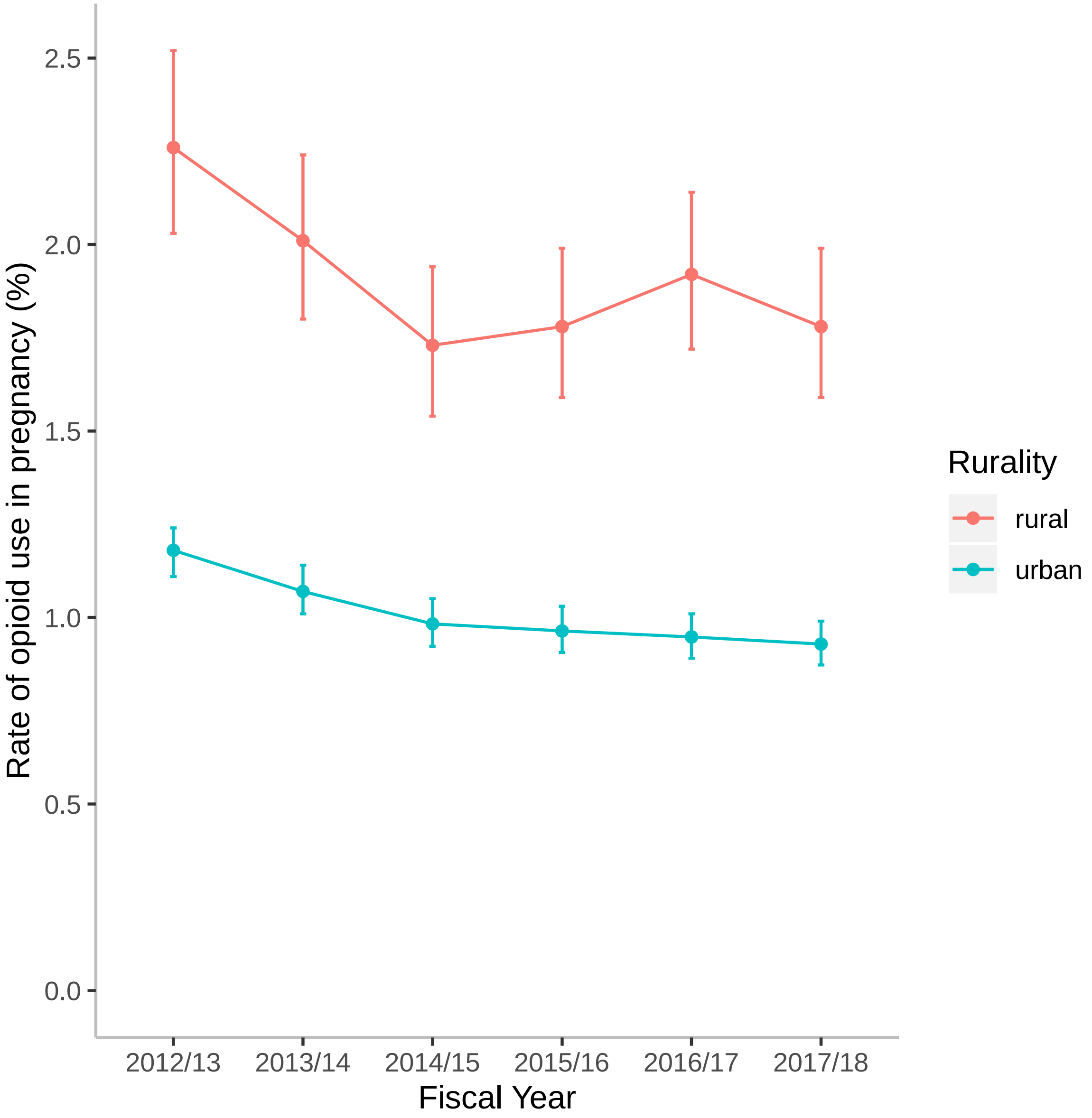

P-linear trend (Urban) < 0.001

P-linear trend (Rural) = 0.006

P-interaction = 0.60

©2020 Corsi DJ et al. *JAMA Network Open*.
